# Specific inhibition of bile acid transport alters plasma lipids and GLP-1

**DOI:** 10.1186/s12872-015-0070-9

**Published:** 2015-07-22

**Authors:** Mats Rudling, Michael Camilleri, Hans Graffner, Jens Juul Holst, Leif Rikner

**Affiliations:** Department of Endocrinology, Metabolism and Diabetes, Metabolism Unit, Center for Endocrinology, Metabolism, and Diabetes, Karolinska Institute at Karolinska University Hospital Huddinge, S-141 86 Stockholm, Sweden; Department of Medicine, and Molecular Nutrition Unit, Karolinska Institute at Karolinska University Hospital Huddinge, S-141 86 Stockholm, Sweden; Department of Biosciences and Nutrition, Karolinska Institute at Karolinska University Hospital Huddinge, S-141 86 Stockholm, Sweden; Mayo Clinic, Rochester, MN USA; Albireo, Göteborg, Sweden; NNF Center for Basic Metabolic Research, the Panum Institute, University of Copenhagen, Copenhagen, Denmark

**Keywords:** Dyslipidemia, Elobixibat, Glucagon-like peptide-1 (GLP-1), Ileal bile acid (BA) transporter (IBAT) inhibitor

## Abstract

**Background:**

Elobixibat is a minimally absorbed ileal bile acid (BA) transporter (IBAT) inhibitor in development against chronic constipation (CC) and constipation-predominant Irritable Bowel Syndrome (IBS-C). CC is associated with an increased risk for cardiovascular disease and type2 diabetes mellitus. The objectives of this study were to evaluate metabolic effects of elobixibat. Effects on plasma lipids and BA synthesis were evaluated utilizing a 4-week, placebo-controlled study in patients with dyslipidemia while changes of glucagon-like peptide-1 (GLP-1) by elobixibat was assayed in samples from a 14 day high-dose elobixibat study in patients with CC.

**Methods:**

Thirty-six dyslipidemic patients, 21 females, mean age 63 years, were randomized to 2.5 mg or 5 mg elobixibat or placebo once daily for four weeks. The primary endpoint was the change in low density lipoprotein (LDL) cholesterol. Secondary endpoints included other lipid parameters and serum 7α-hydroxy-4-cholesten-3-one (C4), a marker of BA (bile acid) synthesis. Another study, in 36 patients with CC treated with high dose elobixibat; 15 mg or 20 mg/day or placebo for 14 days, was evaluated for changes in GLP-1.

**Results:**

In the dyslipidemia study LDL cholesterol was reduced by 7.4 % (*p* = 0.044), and the LDL/HDL ratio was decreased by 18 % (*p* = 0.004). Serum C4 increased, indicating that BA synthesis was induced. No serious adverse events were recorded. In the CC study, GLP-1 increased significantly in both the 15 mg (20.7 ± 2.4 pmol/L; *p* = 0.03) and the 20 mg group (25.6 ± 4.9 pmol/L; *p* = 0.02).

**Conclusions:**

Elobixibat reduces LDL cholesterol and LDL/HDL ratio and increase circulating peak GLP-1 levels, the latter in line with increased intestinal BA mediated responses in humans.

**Trial registrations:**

ClinicalTrial.gov: NCT01069783 and NCT01038687.

## Background

A key step in bile acid (BA) homeostasis is the active uptake of BAs through the apical enterocyte membranes of the distal ileum by the ileal apical sodium-dependent bile acid transporter (IBAT or ASBT [apical sodium-dependent BA transporter]). Inhibition of IBAT reduces the active ileal absorption of BAs, increasing the content of BAs in the colon, thereby stimulating colonic secretion and motility [1, 2]. Studies of one such inhibitor (elobixibat, previously A3309), have proven beneficial in chronic constipation (CC) [3–5]. In addition to their role as detergents facilitating dietary lipid absorption, BAs modulate various metabolic events after binding to specific BA receptors such as the farnesoid x receptor (FXR) and the G-protein-coupled receptor TGR5 [6, 7]. Thus, BAs regulate glucose and lipid metabolism as well as energy expenditure [6].

When ileal BA absorption decreases, fecal loss of BAs increases resulting in an upregulation of hepatic BA synthesis to maintain BA homeostasis. BA synthesis can be monitored from the level of C4 (7α-hydroxy-4-cholesten-3-one) in serum, a marker for the enzymatic activity of cholesterol 7α-hydroxylase [8], the rate-limiting enzyme in BA synthesis. Cholesterol is precursor in the synthesis of BAs. Induction of BA synthesis depletes hepatic cholesterol stores, which is compensated for through increased hepatic synthesis of cholesterol in combination with an increased number of hepatic low density lipoprotein (LDL) receptors that subsequently reduce plasma LDL cholesterol levels. How BAs modulate glucose homeostasis has lately gained renewed interest with studies claiming increased release of glucagon-like peptide-1 (GLP-1) from the intestine following administration with a bile acid binding sequestrant [9] and after rectal administration of taurocholate [10]. Also the mode of action of the hypoglycemic agent metformin has been suggested to include decreased intestinal bile acid absorption [11–13].

Elobixibat is a selective and partial inhibitor of IBAT with a novel and unique mechanism of action. After oral administration, systemic exposure is minimal [14]. In three studies in patients with CC [3–5], elobixibat enhanced colonic transit, thereby improving symptoms of CC such as increased stool frequency concomitant with reduced straining, bloating and hard stools.

The aim of the present investigation was to evaluate metabolic responses of elobixibat. For this purpose, the level of BA synthesis and serum lipid profiles were monitored in dyslipidemic patients treated with elobixibat for 28 days. In addition, we also evaluated whether such treatment may increase serum levels of the incretin GLP-1 in a study of CC patients treated with elobixibat at high dose.

## Methods

### The Dyslipidemia study

#### Study design, baseline eligibility, randomization and medication

This was a single-center, randomized, parallel-group, double-blind, placebo-controlled, two dose, pharmacodynamic study evaluating the effects of elobixibat in dyslipidemic patients. Dyslipidemia was defined as having serum total cholesterol levels >5.5 mmol/L (213 mg/dL). The study was approved by the Linköping University Regional Ethics Review Board. It was performed from February 2010 (first signed informed consent) to July 2010 (last patient visit).

After informed consent was obtained, patients entered a 14 day screening period when routine blood tests, urinalysis, and pregnancy tests were made and demographic data collected. If screening assessments provided proof of eligibility, the patient was randomized. Patients were randomized to receive one tablet daily containing 2.5 mg elobixibat, 5 mg elobixibat or matching placebo for 28 consecutive days. A randomization list was generated by an independent statistician prior to the study and the assignments were concealed from all study personal until the study was closed and the final data transmitted to the study statistician for analysis. All clinical and laboratory study personnel were blinded throughout the study until data were locked and analyzed. Safety was monitored throughout the study. At Day 14 of the treatment period, a visit to document health status, adverse events (AEs) and for blood sampling took place and at the end of the treatment period a similar visit was scheduled. Fourteen days after last day of treatment, a follow-up visit was conducted.

#### Study medication

The IBAT Inhibitor elobixibat is a small molecule known to be a partial inhibitor of the ileal BA transporter (IBAT). The effects of elobixibat on GI motility and fecal output show an increase in fecal output identified at the lowest dose (1 mg/kg) tested [14].

Orally administered elobixibat is minimally absorbed from the GI tract with picomolar levels found in plasma, an expected half-life (t½) in humans of <4 h and no metabolites detectable in human plasma. Given its low bioavailability in plasma, it is expected that its action reflects local inhibition of IBAT. In a multiple ascending dose study (0.1–10 mg), administration of elobixibat was found to be safe and tolerable across the dose levels tested with no serious adverse events or discontinuations [4] and in two Phase II studies in patients with CC, dose levels of 5–10–15–20 mg have been evaluated with beneficial effects on stool frequency, stool consistency and symptoms of CC [3, 5].

#### Study participants

Eligible patients were men or non-pregnant women, 18–80 years of age with a serum cholesterol of >5.5 mmol/l. The body mass index ranged 18.5 up to 35. Major exclusion criteria were: Known cardiovascular disease, s-cholesterol > 8.5 mmol/l, s-triglycerides > 4.0 mmol/l, use of drugs known to alter lipids and/or BAs within 12 weeks of screening visit, patients meeting the criteria for diagnosis of IBS-D by Rome III standards [15] or had any IBS associated symptoms and/or reported loose (mushy) or watery stools during the 12 weeks prior to the screening visit; exception being short term (<5 days) of gastroenteritis.

#### Outcome measures

The primary end point of the study was the change in LDL (low density lipoprotein) cholesterol from baseline to day 28 of treatment. Secondary efficacy end points included changes from baseline in total cholesterol, HDL (high density lipoprotein) cholesterol, LDL/HDL ratio and total triglycerides to Day 14 and to Day 28 of treatment and C4 evaluation was used as the pharmacodynamic marker of bile acid metabolism. The safety end points included changes from baseline in the nature, incidence and severity of AEs, clinical laboratory abnormalities and changes over time in these parameters, vital signs, ECG and physical findings.

#### Statistical methods

An intention to treat (ITT) analysis of all randomized subjects was used as the primary analysis. Patients were randomized in a 1:1:1 ratio to the treatment groups. For the primary end point, the sample size selected yielded a power of 80 % with a least significant difference between treatment groups for LDL cholesterol of 0.4. All efficacy end points were analyzed using the Wilcoxon rank sum test and a two-sided *p*-value of <0.05 was considered significant. No adjustment for multiplicity was performed.

#### Laboratory analyses

Conventional safety laboratory parameters including serum glucose and liver function tests were evaluated in both studies. No difference between the active groups and placebo were identified. Assay of plasma lipoproteins LDL, HDL and total cholesterol and total triglycerides were made using routine clinical methods.

Samples for fasting serum C4 were taken at approximately 8 am and measurements were conducted using high-performance liquid chromatography, as previously described [8].

### The chronic constipation study

#### Study design, baseline eligibility, randomization, medication

The demographics and the results pertaining to efficacy in CC and the safety of elobixibat at high dose levels 15 mg and 20 mg/day have been reported [5]. In brief, 36 female patients with CC were included in a 14 day treatment study to evaluate colonic transit and constipation, 15 mg, 20 mg/day or placebo were administered for 14 days. Serum samples were obtained at baseline, in the morning Day 12 and throughout Day 12 (every 30 min for 2 h and then every hour up to 480 min after drug administration). Patients were administered standardized meals; breakfast immediately after the baseline sample, and a light lunch four hrs after baseline sampling. Total GLP-1 concentrations in plasma were measured by radioimmunoassay after extraction of plasma with 70 % ethanol (vol/vol, final concentration) using antiserum 89390 which has an absolute requirement for the intact amidated carboxy-terminus of GLP-1 7–36 amide and crossreacts fully with GLP-1 9–36 amide, the primary metabolite of dipeptidyl-peptidase −4 mediated degradation. The sum of the two components (total GLP-1 concentration) reflects the rate of secretion of the L-cell. Sensitivity was below 1 pmol/l, and intra-assay coefficient of variation below 5 % [16] (16).

Results are reported as means ± standard error of the mean (SEM) unless otherwise stated. For the analysis of GLP-1, ANCOVA or ANOVA were used depending on whether response or difference vs baseline were evaluated. Comparisons were made between fasting value at start of treatment to fasting value on Day 12 of treatment and comparison of maximum (peak) value on Day 12 *vs.* at treatment allocation with fasting value before treatment as covariate. Values of the area under the curve (AUC) of serum GLP-1 concentrations were calculated using the trapezoidal rule to assess the integrated response of GLP-1 over 480 min. Statistical analyses are based on t-test/Wilcoxon as indicated in the Figures. Reporting of the studies conform to CONSORT-revised and the EQUATOR guidelines [17].

## Results

### Results of the dyslipidemia study

#### Patient flow and follow-up

**Fig. 1:**
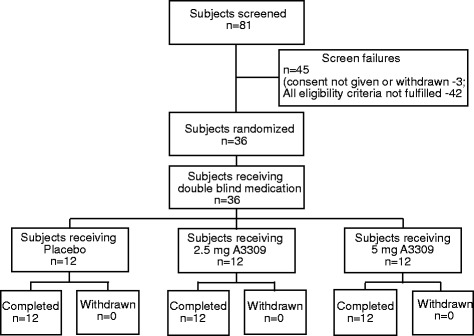
Patient flow chart in the Dyslipidemia study

The study flow and patient demographics is shown in Fig. 1. Out of 81 screened subjects, 36 patients fulfilled eligibility criteria, entered the study and were randomized to treatment with elobixibat or placebo. All patients completed the study; the ITT and the Safety population include the same patients. At baseline, the demographic parameters were similar between the treatment groups (Table 1A). Patients had a mean age of 63 years (range 34–80) and there was a slight preponderance for women (58 %). Baseline lipid profiles were similar across the groups (Table 1A). No changes in safety laboratory values, including liver enzymes and plasma glucose, were identified during the study.

**Table 1:** Demographics and baseline parameters

A. Dyslipidemia study				
		Placebo (*N* = 12)	Elobixibat 2.5 mg (*N* = 12)	Elobixibat 5 mg (*N* = 12)
Female	n (%)	8 (66.7)	7 (58.3)	6 (50.0)
Caucasian	n (%)	12 (100.0)	12 (100.0)	12 (100.0)
Age (years)	Mean (SD)	64.4 (10.1)	60.9 (13.2)	62.7 (11.4)
Weight (kg)	Mean (SD)	76.2 (16.3)	78.1 (16.4)	76.6 (12.8)
BMI (kg/m^2^)	Median	24.8	26.3	26.4
LDL-Cholesterol (mmol/L)	Mean (SD)	4.29 (0.71)	4.61 (0.63)	4,78 (0.65)
Cholesterol (mmol/L)	Mean (SD)	6.38 (0.75)	6.44 (0.57)	6.57 (0.72)
HDL-Cholesterol (mmol/L)	Mean (SD)	1.53 (0.49)	1.34 (0.43)	1.23 (0.30)
LDL/HDL-Cholesterol (mmol/L)	Mean (SD)	3.00 (0.92)	3.77 (1.60)	4.32 (1.73)
B. The CC study				
		Placebo (*N* = 13)	Elobixibat 15 mg (*N* = 12)	Elobixibat 20 mg (*N* = 11)
Female	n (%)	13 (100.0)	12 (100.0)	11 (100.0)
Caucasian	n (%)	11 (92.3)	12 (100.0)	12 (100.0)
Age (years)	Mean (SD)	47.2 (9.30)	38.3 (8.18)	46.1 (6.36)
Weight (kg)	Mean (SD)	70.2 (9.7)	72.9 (15.5)	75.0 (17.0)
BMI (kg/m^2^)	Median	25.9	24.8	26.0
GLP-1 (pmol/L)	Mean (SD)	18.45^a^ (8.63)	15.08 (5.38)	14.67^b^ (5.92)

^a^ *n* = 11

^b^ *n* = 9

#### Effects of elobixibat on lipid profiles and on C4

**Fig. 2:**
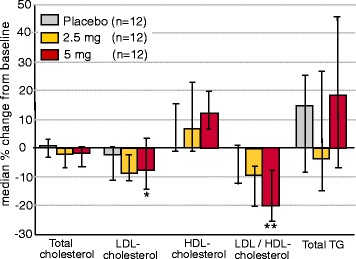
Median % changes (interquartile 25–75 % range) from baseline for plasma total cholesterol, LDL, HDL, LDL/HDL ratio and triglycerides (TG) in the Dyslipidemia study. *: *p* = 0.044 **: *p* = 0.004 (Wilcoxon)

There was a significant (*p* = 0.044) change from baseline to end of treatment in LDL cholesterol (7.4 %) for the 5 mg elobixibat treatment group – the primary end point of the study (Fig. 2). No significant change was achieved for the 2.5 mg elobixibat cohort. When analyzing the mean and median change values from baseline to Days 14 and 28, there was a more prominent reduction in the drug-treated groups as compared to placebo whereas on day 42 (14 days after cessation of treatment) there was no clear difference.

Elobixibat treatment did not significantly alter total cholesterol, HDL cholesterol or triglycerides (Fig. 2). In contrast, the LDL/HDL ratio was significantly reduced on Day 28 in the 5 mg group (*p* = 0.004) (18 %) whereas no effect was seen in the 2.5 mg group (p = 0.06) (Fig. 2).

**Fig. 3:**
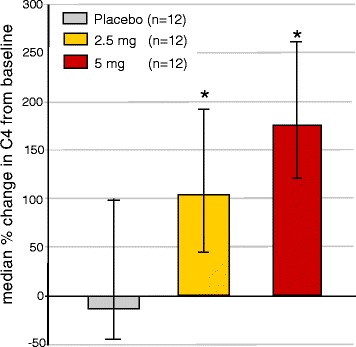
Median % changes (interquartile 25–75 % range) from baseline for plasma C4 in the Dyslipidemia study. *: *p* <0.03 (Wilcoxon)

C4 was significantly increased in both the 2.5 mg and in the 5 mg elobixibat groups on Day 28 (*p* = 0.03 for both groups) (Fig. 3).

#### Safety assessments

No serious adverse events (SAEs) were reported during the dyslipidemia study. AEs were most prevalent in the placebo group (92 % of the patients reported 27 AEs). In the 2.5 mg and 5 mg treatment groups, 50 % and 67 % of the patients reported a total of 11 and 14 AEs respectively. Most of the AEs were recorded as mild and transient and only four were moderate, two in the placebo group and two in the 5 mg group; none of them were judged to be related to the study drug. No patient discontinued the study. Table 2 outlines the incidence of AEs. The most common AEs were headache, diarrhea and constipation. Notably, diarrhea was reported in 3 patients on placebo, 2 patients on 2.5 mg and 3 patients on 5 mg.

**Table 2:** Adverse events occurring in more than one subject in the Dyslipidemia study. No serious adverse events and no discontinuations were identified

		Placebo (*N* = 12)	A3309 - 2.5 mg (*N* = 12)	A3309 - 5 mg (*N* = 12)
MedDRA system organ class	MedDRA preferred term	n (%)	n (%)	n (%)
Gastrointestinal disorders	Abdominal distension	1 (8.3)	1 (8.3)	1 (8.3)
	Constipation	4 (33.3)	1 (8.3)	-
	Diarrhoea	3 (25.0)	2 (16.7)	3 (25.0)
General disorders and administration site conditions				
	Pyrexia	1 (8.3)	-	1 (8.3)
Infections and infestations				
	Pharyngitis	1 (8.3)	-	1 (8.3)
	Rhinitis	2 (16.7)	-	-
Musculoskeletal and connective tissue disorders	Myalgia	2 (16.7)	-	-
Nervous system disorders	Dizziness	2 (16.7)	-	-
	Headache	3 (25.0)	3 (25.0)	3 (25.0)
Renal and urinary disorders	Polyuria	1 (8.3)	1 (8.3)	-
Respiratory, thoracic and mediastinal disorders	Cough	1 (8.3)	-	1 (8.3)

### Results of the CC study

Demographic parameters are described in Table 1B. Efficacy on pharmacodynamics and clinical endpoints of CC and safety assessments have been reported previously [5].

No changes in safety laboratory values, including liver enzymes and plasma glucose, were identified during the study.

#### Effects of elobixibat on circulating GLP-1 levels in constipated patients

**Fig. 4:**
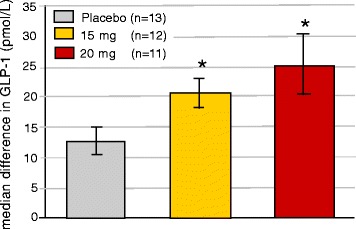
GLP-1 (pmol/L) in the CC study. Data shown are the difference between peak values Day 12 and the basal pre-study value. Mean ± SEM. *: *p* < 0.05 (Wilcoxon)

Peak values for GLP-1 levels increased significantly during the day, when adjusted for baseline values, for both the 15 mg (20.7 ± 2.4 pmol/L) and the 20 mg (25.6 ± 4.9 pmol/L) groups (*p* = 0.03 and *p* = 0.02 respectively) compared with placebo (12.8 ± 2.4 pmol/L) (Fig. 4). Peak values were observed at lunch time. No significant differences were observed for the basal morning levels at Day 12 of treatment when compared to levels prior to treatment. Similarly, there were no significant differences in total AUC levels during the 480 min period (201 ± 12 pmol/L*hr (15 mg), 225 ± 27 pmol/L*hr (20 mg) and 180 ± 27 pmol/L*hr (placebo).

## Discussion

The current results provide insight in metabolic responses obtained when administering an IBAT inhibitor to dyslipidemic patients with elevated cholesterol levels, and when employed at high dose to patients with CC. At a dose of 5 mg elobixibat reduced LDL cholesterol by 7.4 % and decreased the LDL/HDL ratio by 18 %. The reduced LDL/HDL ratio is of particular interest since it is an important risk factor for the development of cardiovascular disease (CVD) [18]. The beneficial effects of elobixibat on serum lipids confirm previous findings by Chey et al. [3]. A limitation of this paper is the fact that data have been collected from two different populations (patients with dyslipidemia or with chronic constipation). It would have been of interest to assess GLP-1 in patients with dyslipidemia as well and to perform a thorough analysis of subfractions of LDL and HDL but given the set-up of the study, this was not possible due to restrictions in the sampling.

Although no patient discontinued the study, signs of increased colonic transit were identified (Table 2). On the other hand, the improved LDL/HDL ratio should be beneficial in patients with CC. This because it has been reported that constipation is a risk factor for CVD events [19] as shown from women with severe constipation having a 23 % higher risk for CVD events, and an unadjusted risk of death due to CVD almost five-fold higher: 3.25 (95 % CI 1.76–5.01) in severe constipation as compared to 0.76 (95 % CI 0.54–1.06)/1000 person-years in subjects without constipation. Moreover, Salmoirago et al. [19] reported that the more severe state of constipation at baseline, the more frequent is the use of cholesterol-lowering medication. Further support for a relation between atherosclerosis and constipation was found in a study [20] on patients with known CVD having a 2.5-fold higher prevalence of constipation as compared to subjects without constipation. Cholesterol is to a great extent eliminated from the body through fecal loss of BAs [21] (21) that is clearly reduced in patients with CC [22–24]. Interestingly, also in patients with CVD, fecal BA excretion is significantly reduced as compared with non-CVD patients [25].

Considering the above findings, it is conceivable that an IBAT inhibitor like elobixibat administered for the treatment of CC may be particularly attractive not only to relieve symptoms of constipation but also to lower LDL cholesterol and reduce the increased risk for CVD observed in subjects with constipation.

The significantly increased levels of GLP-1 in response to a dose level of elobixibat approximately twice the target dose for patients with CC suggests that IBAT inhibition stimulates intestinal L cells, presumably through elevated BAs in the intestine interacting with TGR5 receptors, thereby stimulating synthesis and secretion of GLP-1 [6, 7, 9, 26, 27]. As compared to treatment with DPP-4 inhibitors, the meal-induced plasma GLP-1 levels with elobixibat treatment are smaller [28]. However, it is important to point out that the meal stimulus in this study was a standardized low calorie chicken breast lunch, and that GLP-1 AUC measurements were evaluated over a longer time period, most likely inducing a lower response than a high calorie load or an oral glucose tolerance test.

The most commonly used drug in T2DM, metformin, probably in part acts through enhancing GLP-1 secretion [28]. This effect may be mediated through IBAT inhibition since metformin is known to suppress active BA absorption from the ileum [11]. Although data are not uniform [29] other IBAT-inhibitors have demonstrated incretin-releasing properties in animals [30]. Intriguingly, BA sequestrants like cholestyramine bind BAs and therefore reduce free BAs in the colon, have been reported beneficial in T2DM [31]. Results have shown that colesevelam when bound to BAs may activate TGR-5 receptors in vitro [10]. On the other hand, in healthy humans cholestyramine-induced changes of serum glucose can appear independent of serum GLP-1 levels (Rudling M, unpublished research).

The positive effects of IBAT-inhibition on GLP-1 secretion may thus be particularly beneficial in CC patients with T2DM or pre-diabetes; two disease entities associated with increased risk for constipation [18, 32, 33].

## Conclusion

In summary, the studies reported here provide evidence of decreased LDL cholesterol and increased GLP-1 levels when using elobixibat in patients with dyslipidemia and with CC respectively; these effects are consistent with metabolic effects of BAs in the ileocolonic region in humans. Further development of IBAT-inhibitors to combat metabolic disease will possibly be restricted by the effects of IBAT inhibition on colonic transit, resulting in diarrhea. However, given the association between symptoms of constipation with cardiovascular disease and T2DM, it seems that an IBAT inhibitor like elobixibat, in addition to positive effects on symptoms and signs of constipation, may provide positive metabolic side effects reducing the risk for CVD and T2DM.

## Abbreviations

ASBT: Apical sodium-dependent BA transporter
BA: Bile acids
C4: 7α-hydroxy-4-cholesten-3-one
CC: Chronic constipation
FXR: Farnesoid X receptor
GLP-1: Glucagonlike peptide 1
HDL: High density lipoprotein
IBAT: Ileal bile acid transporter
IBS-C: Constipation-predominant irritable bowel syndrome
HDL: High density lipoprotein
T2DM: Type2 diabetes mellitus
